# Near-Real-Time Integration of Multi-Source Seismic Data

**DOI:** 10.3390/s26020451

**Published:** 2026-01-09

**Authors:** José Melgarejo-Hernández, Paula García-Tapia-Mateo, Juan Morales-García, Jose-Norberto Mazón

**Affiliations:** Department of Software and Computing Systems, Universidad de Alicante (UA), 03690 Alicante, Spain; jose.melgarejo@ua.es (J.M.-H.); paula.garcia@ua.es (P.G.-T.-M.); juan.morales@ua.es (J.M.-G.)

**Keywords:** seismic data integration, near-real-time systems, open APIs, FAIR principles

## Abstract

The reliable and continuous acquisition of seismic data from multiple open sources is essential for real-time monitoring, hazard assessment, and early-warning systems. However, the heterogeneity among existing data providers such as the United States Geological Survey, the European-Mediterranean Seismological Centre, and the Spanish National Geographic Institute creates significant challenges due to differences in formats, update frequencies, and access methods. To overcome these limitations, this paper presents a modular and automated framework for the scheduled near-real-time ingestion of global seismic data using open APIs and semi-structured web data. The system, implemented using a Docker-based architecture, automatically retrieves, harmonizes, and stores seismic information from heterogeneous sources at regular intervals using a cron-based scheduler. Data are standardized into a unified schema, validated to remove duplicates, and persisted in a relational database for downstream analytics and visualization. The proposed framework adheres to the FAIR data principles by ensuring that all seismic events are uniquely identifiable, source-traceable, and stored in interoperable formats. Its lightweight and containerized design enables deployment as a microservice within emerging data spaces and open environmental data infrastructures. Experimental validation was conducted using a two-phase evaluation. This evaluation consisted of a high-frequency 24 h stress test and a subsequent seven-day continuous deployment under steady-state conditions. The system maintained stable operation with 100% availability across all sources, successfully integrating 4533 newly published seismic events during the seven-day period and identifying 595 duplicated detections across providers. These results demonstrate that the framework provides a robust foundation for the automated integration of multi-source seismic catalogs. This integration supports the construction of more comprehensive and globally accessible earthquake datasets for research and near-real-time applications. By enabling automated and interoperable integration of seismic information from diverse providers, this approach supports the construction of more comprehensive and globally accessible earthquake catalogs, strengthening data-driven research and situational awareness across regions and institutions worldwide.

## 1. Introduction

The continuous monitoring of global seismic activity is fundamental for hazard assessment, early warning, and disaster risk reduction [1,2]. Public institutions such as the *United States Geological Survey* (USGS) (https://earthquake.usgs.gov (accessed on 21 January 2025)), the *European-Mediterranean Seismological Centre* (EMSC) (https://www.emsc-csem.org (accessed on 21 January 2025)), and the *Spanish National Geographic Institute* (IGN) (https://www.ign.es/web/ign/portal (accessed on 21 January 2025)) provide high-quality seismic information through open Application Programming Interfaces (APIs) and public repositories [3,4,5]. These providers play a central role in global seismic monitoring. However, despite their reliability and openness, these catalogs operate independently. They adopt distinct data models and use heterogeneous identifiers and update policies. As a result, the same earthquake is often reported multiple times under different identifiers, magnitudes, or origin times. This redundancy complicates automated data fusion and reduces the consistency of real-time situational awareness. Integrating these heterogeneous data streams can substantially improve the speed and accuracy of earthquake early warning systems. This improvement enables faster alerts, shorter emergency response times, and reduced human and economic losses during seismic crises [2,6].

The lack of consistent data structures, metadata conventions, and access protocols continues to hinder seamless integration into unified and interoperable monitoring frameworks [7,8]. Most infrastructures still rely on centralized acquisition systems or vendor-specific architectures that restrict interoperability and complicate the combination of data from multiple providers [9]. Seismic information is also inherently dynamic. Events are revised frequently, updates occur in near-real time, and networks often report partially overlapping detections. These characteristics demand acquisition systems capable of operating continuously and efficiently with heterogeneous data models [7]. In this context, aligning seismic data integration with the *Findable, Accessible, Interoperable, and Reusable* (FAIR) principles strengthens data governance and scientific reproducibility [8,10]. In particular, ensuring that each recorded event is uniquely identifiable and traceable to its originating institution is essential. Storing events in standardized, machine-readable formats improves both reusability and long-term preservation of seismic knowledge. These practices also facilitate the integration of seismic information into broader environmental and geospatial data ecosystems. This approach supports interoperability with initiatives such as the *European Open Science Cloud* (EOSC) [11] and emerging European Data Spaces [12].

Large-scale infrastructures such as the *Federation of Digital Seismic Networks* (FDSN) [13] have significantly improved the standardized acquisition and dissemination of seismic data through common formats and web services. However, their scope remains focused on data access rather than on cross-institutional integration, as individual networks continue to use distinct metadata schemas, event identifiers, and update policies. As a result, harmonization, deduplication, and validation across catalogs are not addressed at the infrastructure level. They often require ad hoc processing, limiting interoperability and reproducibility.

The framework proposed in this work complements existing seismic sensing infrastructures by addressing the data integration layer. It enables automated aggregation and reuse of seismic event data across institutional and national boundaries. The system operates on event catalogs produced by dense networks of modern ground-motion sensors, including broadband seismometers and strong-motion accelerometers [14]. Rather than replacing existing provider infrastructures, the framework builds upon their published catalogs. A modular and automated design is implemented to unify heterogeneous seismic data sources in near real time. Lightweight containerization and scheduling technologies are employed to acquire, standardize, and persist seismic data with high reliability and scalability. The framework is conceived as a foundation for future interoperability services, enabling deployment as a microservice within open data infrastructures and environmental data catalogs.

The main contributions of this paper are as follows:

1. The design of a modular and containerized architecture for the near-real-time acquisition and harmonization of seismic event data from multiple public APIs (USGS, EMSC, IGN).
2. The implementation of an automated scheduling and validation pipeline that ensures reliable short-term and long-term acquisition windows, maintaining data consistency across heterogeneous catalog formats.
3. A quantitative evaluation of latency, update frequency, geospatial overlapping, and cross-catalog discrepancies, including an empirical analysis of duplicate detections and magnitude harmonization across networks.

## 2. Related Work

This section reviews existing infrastructures, software frameworks, and operational approaches related to the acquisition, dissemination, and integration of seismic data. The focus is placed on large-scale seismological infrastructures, open data platforms, and software systems that support real-time or near-real-time access to earthquake information. Particular attention is given to their scope, interoperability mechanisms, and limitations with respect to cross-provider harmonization. This review provides the context against which the proposed framework is positioned, highlighting the gap addressed by the present work.

The collection and dissemination of seismic data are sustained by a small number of large-scale infrastructures that underpin global seismology. Among them are Seismological Facility for the Advancement of Geoscience (SAGE) (https://www.iris.edu/hq/sage (accessed on 25 January 2025)), the *Global Seismographic Network* (GSN), GEOSCOPE (http://geoscope.ipgp.fr (accessed on 25 January 2025)), and ORFEUS (https://www.orfeus-eu.org/ (accessed on 25 January 2025)), which operate under the FDSN framework. These facilities provide standardized waveform formats and event catalogs through FDSN Web Services, ensuring open and consistent data access. Major agencies such as the USGS, EMSC [4], and the IGN [5] maintain complementary APIs and feeds widely used for real-time reporting and situational awareness [2,3]. Despite formal standardization efforts, data models and metadata conventions remain inconsistent across networks. These inconsistencies affect even basic parameters such as magnitude type, uncertainty fields, and event identifiers [7,9]. This heterogeneity continues to hinder cross-network integration and reliable event identification.

To date, no open platform performs automated harmonization or deduplication across the independent seismic feeds operated under the federated data landscape. This limitation exposes a persistent lack of interoperability that extends beyond seismology. In the broader domain of Earth and environmental data, several initiatives promote interoperable and FAIR-compliant frameworks for cross-domain integration, including INSPIRE (https://inspire.ec.europa.eu/ (accessed on 25 January 2025)) and the European Open Data Portal [15]. These initiatives promote the adoption of standardized APIs, persistent identifiers, and shared vocabularies, thereby supporting data discoverability and reuse. However, seismological networks remain weakly connected to these infrastructures, and their information still depends on heterogeneous metadata schemes and isolated repositories. This fragmentation limits interoperability with hydrological, meteorological, and geotechnical datasets and constrains the role of seismic data in multi-hazard analysis and digital-twin initiatives [16,17]. Addressing this limitation requires not only metadata harmonization but also operational mechanisms capable of unifying real-time data across providers.

In the European context, the European Plate Observing System-European Research Infrastructure Consortium (EPOS-ERIC) represents the main research infrastructure dedicated to solid Earth sciences [16]. It provides unified access to seismic data, products, and services through the EPOS Data Portal in a FAIR-compliant manner. EPOS integrates earthquake catalogs and related products from providers such as ORFEUS, EMSC, and national agencies including the IGN. While EPOS primarily focuses on large-scale federation and long-term data management within Europe, it does not address the automated harmonization and deduplication of near-real-time seismic feeds across independent providers. In contrast, the system proposed in this work targets this operational integration layer, providing a lightweight and automated solution for near-real-time aggregation and cross-catalog comparison.

At the software level, frameworks such as Python Framework for Seismological Data Processing (ObsPy) [8] and Seismological Communication Processor (SeisComP) [18] have become standard tools for accessing and processing seismic data. ObsPy provides flexible waveform retrieval and metadata handling through Python scripting, while SeisComP supports real-time acquisition and event management within individual network deployments. However, both require extensive manual configuration and do not natively support automated cross-network harmonization, deduplication, or integrated quality control workflows. Complementary systems such as IRIS Metrics for Automated Quality Control of Seismic Data (MUSTANG) [19] and ISPAQ [20] focus on network-level performance metrics, but do not address inter-catalog consistency or duplicate event resolution.

Systematic analyses have identified the lack of interoperable metadata standards as a key limitation in modern Earthquake Early Warning Systems (EEWS) [21]. At the same time, Internet-of-Things (IoT)-based warning systems remain fragmented across networks and regions [22], with limited capacity for sharing event information or metadata in a consistent way. This fragmentation exemplifies the broader challenge of integrating heterogeneous seismic data sources under a common operational framework. Open science initiatives such as the EOSC [11] and the European Data Spaces framework [12,23] promote data sharing and interoperability across disciplines. However, their adoption in seismology has largely remained confined to metadata alignment rather than operational data integration [24,25,26]. Together, these limitations highlight the persistent gap between standardized metadata and fully interoperable seismic information systems.

At the systems level, developments in IoT-based environmental monitoring and digital-twin infrastructures have shown that heterogeneous sensor streams can be managed through containerized and event-driven architectures. Technologies such as the OGC SensorThings API [27,28], MQTT [29], and RESTful microservices support scalable, near-real-time interoperability across distributed systems [30]. These approaches provide a mature technological basis for cross-domain data integration, but their implementation in seismology remains limited. These paradigms have also converged with recent advances in software architecture, where containerized API orchestration has gained traction in IoT environments as a scalable solution for managing distributed data endpoints. A representative example is the Docker-based RESTful framework proposed by Mabotha et al. [31], which uses FastAPI and PostgreSQL to dynamically configure and deploy data interfaces. This approach demonstrates how containerization can simplify deployment and improve reproducibility.

The framework presented in this study extends these concepts to seismology, automating retrieval, harmonization, and deduplication across multiple providers (USGS, EMSC, IGN) within a reproducible Docker-based architecture that directly addresses cross-provider integration and operational reproducibility in seismological data management. In contrast to the aforementioned infrastructures and software frameworks, which either focus on data access, network-level processing, or regional federation, the framework presented in this study explicitly addresses cross-provider integration, harmonization, and deduplication as first-class operational objectives.

## 3. Materials and Methods

This section describes the acquisition of heterogeneous seismic data from multiple authoritative sources, the modular Docker-based (version 28.2.2) architecture that automates their ingestion and persistent storage, and the harmonization workflow designed to standardize, validate, and enrich event information for consistent and FAIR-compliant integration.

### 3.1. Data Acquisition and Sources

Seismic event data are periodically collected through automated acquisition cycles in near-real-time, subject to provider update delays. Multiple authoritative providers are integrated to ensure broad geographical coverage and consistent temporal updates. The acquisition layer integrates both public APIs and structured web data extraction, each offering complementary information at different spatial and temporal scales. The integrated sources jointly cover regional, national, and global seismic activity, ensuring comprehensive spatial coverage and redundant event validation across independent catalogs. The main providers are summarized below:

- **USGS**: Provides global earthquake information through a public, unauthenticated *GeoJSON* API fully compliant with the standard specification [32]. Each record includes magnitude, hypocentral coordinates, depth, and origin time, along with auxiliary metadata such as seismic intensity maps (*shakemaps*) and quality indicators when available.
- **EMSC**: Distributes earthquake data for Europe and the Mediterranean region through a public *JSON* endpoint using a *GeoJSON-like* structure. Although it mirrors the geometry/properties layout of the USGS feed, several parameters, like, latitude, longitude, and depth, are redundantly declared outside the geometry object, requiring specific parsing during acquisition. These records complement the global coverage of the USGS feed by providing higher spatial resolution and additional regional detections.
- **IGN**: Publishes seismic activity across Spain and adjacent areas. In the absence of a formal API, data are extracted from a *JavaScript* file embedded within the official web portal, which defines an internal *JSON* object following a simplified *GeoJSON*-like structure. This embedded format must be retrieved and decoded programmatically through pattern matching before parsing, introducing additional heterogeneity in the acquisition process.

Their respective access methods and data conventions are detailed in Table 1, which provides a comparative overview of the main characteristics and data formats adopted by each provider.

Both USGS and EMSC include metadata describing their contributing networks. However, these provenance fields differ in meaning, completeness, and structure. In the USGS feed, the sources field lists the seismic networks involved, while net identifies the primary reporting network code (e.g., us for the national USGS network or ak for the Alaska Earthquake Center). The EMSC catalog provides both the originating authority (auth), typically a national or regional agency, and the catalog tag (source_catalog), indicating the internal EMSC data stream such as *EMSC-RTS*. These provenance indicators are not standardized or cross-referenced. As a result, the same event may appear independently across catalogs without explicit linkage, requiring consistency checks based on temporal, spatial, and magnitude similarity to identify shared detections.

### 3.2. Boundaries and Tectonic Plates Identification

In addition to seismic event data, the system incorporates two external geospatial datasets to determine the country attribution and tectonic plate associated with each earthquake epicenter. Tectonic plate identification is based on the *PB2002* global plate model published by Bird [33]. The dataset is provided as polygonal geometries and ingested into a *PostGIS*-enabled spatial database, where it defines the global distribution of major and minor tectonic plates widely used in seismological and geodynamic studies. National boundaries are derived from the Natural Earth dataset [34], using the *ne_10m_admin_0_countries* layer. This layer provides generalized international country polygons at a global scale under an open-data license and is likewise stored as polygonal data in the same spatial database.

Spatial attribution is performed through point-in-polygon intersection operations executed at the database level. Each earthquake epicenter is represented as a point geometry and intersected against polygonal layers representing tectonic plates and national boundaries using *PostGIS* spatial queries. All geometries are stored and processed in a common geographic coordinate reference system (WGS 84, EPSG:4326). For each epicenter, the first intersecting polygon is selected to assign the corresponding tectonic plate and country attributes.

In cases where an epicenter falls exactly on a polygon boundary or intersects multiple polygons, the first matching polygon returned by the spatial join is retained. Events that do not intersect any national boundary polygon (e.g., offshore events) are stored without a country attribution, while tectonic plate attribution remains available for all events due to the global coverage of the PB2002 model.

### 3.3. System Architecture and Processing Pipeline

**Figure 1:**
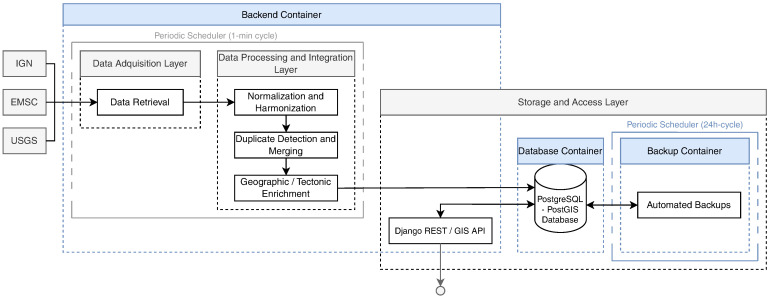
Architecture of the automated acquisition and processing pipeline showing the main *Docker* containers (blue) and functional layers (gray): (i) data acquisition layer for asynchronous retrieval from multiple seismic sources, (ii) data processing and integration layer handling harmonization, deduplication, and geographic enrichment, and (iii) storage and access layer providing persistent geospatial storage and public access via a *PostGIS*-enabled backend.

The system follows a modular and fully automated architecture designed to continuously acquire, normalize, and integrate heterogeneous seismic data from multiple international providers in near real time. Its general structure, illustrated in Figure 1, is implemented within a multi-container *Docker Compose* environment ensuring portability and reproducible deployment across platforms. The system comprises three coordinated services: a backend container running the *Django REST Framework GIS* environment, a *PostgreSQL-PostGIS* database (versions 16.4 and 3.4 respectively) providing persistent geospatial storage, and an auxiliary backup container responsible for automated data preservation and recovery.

Within the backend container, a periodic *cron* scheduler orchestrates the execution of the acquisition and processing workflow, triggering a synchronization cycle every minute to maintain continuous alignment with external seismic data sources. As detailed in Section 4.1, the experimental evaluation confirmed that this design operates stably under continuous conditions, ensuring complete event coverage with minimal latency and sustainable long-term performance.

#### 3.3.1. Data Acquisition Layer

The acquisition layer is responsible for retrieving raw seismic event data from multiple international providers in near real time and preparing them for subsequent harmonization. The process operates continuously through scheduled acquisition cycles. During each cycle, parallel HTTP requests are executed to retrieve data from the USGS, IGN, and EMSC services. Connection reuse and concurrent execution are employed to minimize latency and network overhead. The retrieved data are parsed and preliminarily structured into a unified internal format. This format preserves the original metadata and prepares them for full harmonization during subsequent processing. Each request incorporates timeout handling and error control to ensure resilience against transient network failures and incomplete responses, providing a robust and fault-tolerant acquisition workflow.

The system maintains an internal synchronization state that distinguishes between initial and incremental updates, enabling it to adjust the temporal window of each acquisition cycle dynamically. During the first execution, when no prior synchronization state or local records exist, a full initialization is performed by retrieving recent historical data to populate the catalog. Specifically, the most recent 30 days of seismic events are requested for the USGS and EMSC sources, while a shorter three-day window is used for the IGN due to intrinsic limitations of its data access mechanism and the absence of an official API. Subsequent cycles automatically request only the events generated within the last 24 h. This synchronization state is persistently stored and updated after each acquisition cycle. As a result, redundant downloads are prevented and previously synchronized data are not reprocessed. This mechanism ensures continuous alignment with global seismic activity and provides a consistent and up-to-date data stream for downstream processing.

#### 3.3.2. Data Processing and Integration Layer

To ensure that all incoming seismic records conform to a consistent and interoperable schema, the system performs harmonization, validation, and enrichment of the parsed data during each acquisition cycle. The harmonization module converts the heterogeneous structures of the original feeds into a unified schema consistent with Table 1, resolving differences among providers in field names, coordinate conventions, and temporal formats. All temporal values are expressed in UTC. Depth measurements are standardized to positive values in kilometers, correcting the negative-depth convention used by some providers. In accordance with the FAIR data principles, each record is assigned a persistent global identifier. This identifier is generated as a hash-based value derived from the original source and event identifier, ensuring full traceability and reproducibility across catalogs.

Once normalized, the events are enriched with geographic and tectonic context through spatial operations performed in *PostGIS*. The epicentral coordinates of each event are intersected with polygonal layers representing national boundaries and global tectonic plates to determine the country of origin and the corresponding tectonic plate. When available, USGS intensity maps are also retrieved to estimate the extent of potentially affected regions, complementing the tectonic and geographic enrichment process. All spatial operations are executed automatically within the same processing cycle, ensuring that each stored event includes complete and internally consistent spatial metadata.

Finally, a deduplication stage identifies equivalent detections reported by multiple providers within predefined temporal, spatial, and magnitude windows. Events are cross-compared based on origin time, epicentral distance, and magnitude difference. Redundant entries are linked to a single canonical record, preserving both provenance and consistency within the harmonized catalog. Detected duplicates are not discarded. Instead, they are explicitly linked through persistent duplicate records that retain their respective temporal (Δt), spatial (Δd), and magnitude (ΔM) differences, enabling subsequent quality assessment and statistical validation. The operational thresholds adopted for this automatic duplicate detection (Δt≤8s, Δd≤8km, ΔM≤0.7) were empirically derived from the inter-catalog variability analysis described in Section 4.3, which quantifies the natural discrepancies observed among independent sources. This procedure maintains the integrity of the unified event repository, minimizes redundancy, and ensures that cross-source detections are consolidated in near real time while retaining the original event provenance.

#### 3.3.3. Storage and Access Layer

The harmonized and enriched event catalog is stored in a *PostgreSQL-PostGIS* database that provides persistent geospatial storage, spatial indexing, and efficient query performance for large datasets. This database serves as the central repository of the system, ensuring long-term accessibility and reproducibility of all processed seismic events and metadata. An independent backup container performs scheduled database dumps to preserve data integrity and enable recovery in case of failure or for long-term archiving.

The main backend, implemented using the *Django REST Framework GIS*, exposes the unified catalog via a *GeoJSON*-based REST API that provides structured and interoperable access to the harmonized data. The API supports attribute-based filtering, full-text search, and bounding-box spatial queries, enabling external applications to dynamically query and retrieve earthquake records based on spatial and temporal constraints.

## 4. Results

This section presents the operational validation and performance results of the proposed framework, including ingestion performance metrics, temporal behavior under continuous operation, and cross-source harmonization analyses.

### 4.1. Operational Validation and Performance Assessment

**Figure 2:**
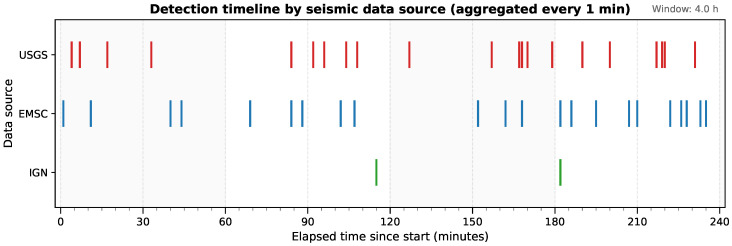
Detection timeline for the USGS (red), EMSC (blue) and IGN (green) seismic data sources during a representative 4 h window of the 24 h acquisition test. Each vertical mark represents an aggregated detection (1 min resolution).

To assess temporal behavior and ingestion reliability, a controlled monitoring experiment was conducted with the three integrated data providers. During this validation phase, retrieval cycles were temporarily reduced to 3 s. This configuration was used to evaluate the upper performance limits of the acquisition framework and capture fine-grained temporal dynamics. Both newly published events and repeated detections were continuously logged for all sources. Figure 2 illustrates the temporal distribution of event arrivals during a representative four-hour window, where each mark corresponds to the instant when a provider reported one or more previously unseen seismic events. The resulting timeline reveals an asynchronous yet stable flow of information, characterized by short bursts and idle intervals that reflect the heterogeneous update schedules of each provider.

To complement this short-term inspection, the acquisition routine was operated continuously for 24 h. An initial synchronization cycle was executed and excluded from performance statistics in order to evaluate long-term stability and throughput under sustained operation. Table 2 summarizes the per-source metrics for this extended run, including the number of new events and updates, the average event rate, and the mean *publication delay*. In this context, publication delay is defined as the time elapsed between the earthquake origin time and the most recent version of that event ingested by the system during the observation window. Because seismic events are frequently revised after their initial release, this metric reflects cumulative latency up to the last observed update. It therefore differs from time to first publication. Given the strongly right-skewed distribution of update times, publication delays are summarized using the median rather than the mean. Across all sources, events reached their latest ingested state after a median delay of 29.1 min, with per-source medians ranging from 20.73 min for EMSC and 29.37 min for USGS to 37.16 min for IGN. Despite substantial dispersion driven by late revisions of a small number of events, all three providers maintained nearly perfect availability (>99.99%). Ingestion latency remained consistently below one second across all sources.

### 4.2. Long-Term Acquisition Performance

To complement the short-term high-frequency validation described in Section 4.1, a second and independent experiment was conducted to evaluate sustained operation under realistic conditions. The 24 h experiment served as a stress test to characterize ingestion dynamics and upper performance limits. In contrast, the long-term deployment focuses on steady-state behavior, availability, and robustness during continuous autonomous operation.

Table 3 summarizes the acquisition performance over the seven-day period, excluding the initial synchronization cycle. During this synchronization phase on a clean deployment, the system executed an initialization procedure in which recent historical data were retrieved to populate the local catalog. This process required approximately 11.3 min. It resulted in the ingestion of 22,092 pre-existing seismic events, including 9382 from the USGS, 12,666 from the EMSC, and 44 from the IGN. These differences reflect provider-specific initialization windows. Following this initialization, all acquisition cycles operated exclusively in incremental mode. Throughout the ensuing seven-day steady-state period, availability remained at 100% for all providers, confirming uninterrupted acquisition and stable synchronization. During this interval, the system ingested 4533 newly published seismic events, processed 3733 subsequent updates, and identified 595 duplicated detections across sources as part of its normal operational workflow.

A comparison between the high-frequency 24 h experiment (Table 2) and the seven-day steady-state deployment (Table 3) indicates that reducing the polling interval from 3 s to 60 s does not introduce observable inconsistencies in catalog completeness or system synchronization. While the 24 h experiment characterizes update dynamics under intensive polling, the seven-day deployment demonstrates that the system maintains stable ingestion behavior when operating with a one-minute acquisition cycle. Under this configuration, all publicly released events and subsequent revisions were incorporated during sustained operation, with no indications of ingestion failures or inconsistencies in the resulting unified catalog.

It is important to note that the *Duplicated Events* column refers to events identified as secondary representations of an already existing canonical event detected by another source. For each group of coincident detections, a single event is designated as the canonical record, while additional detections from other providers are stored as duplicates linked to that canonical event. Consequently, a source may report zero duplicated events while still contributing canonical events that are duplicated by other providers. For example, during the seven-day deployment, most duplicated detections correspond to events originally reported by the USGS or the IGN and subsequently detected by the EMSC. This explains why duplicated counts are concentrated in the latter.

**Figure 3:**
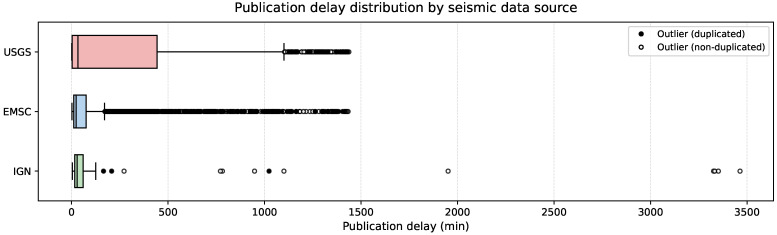
Distribution of publication delay for the USGS (red), EMSC (blue), and the IGN (green) sources. Outliers are represented as circular markers: filled black circles correspond to outliers associated with duplicated events (either canonical or duplicate records), while hollow circles indicate non-duplicated outliers.

By contrast, the publication delay profiles differ substantially among sources, as illustrated in Figure 3. The USGS exhibits the highest median publication delay (33.68 min), reflecting longer cumulative revision intervals on average. In contrast, the IGN is characterized by the largest dispersion in delay values. This high variability is driven by a small number of events with exceptionally long revision intervals, with extreme values reaching up to 3463.86 min. These extreme values correspond to a very small fraction of events. They do not affect the median publication delay, which remains below one hour for all sources. Overall, the elevated variability observed for the IGN reflects heterogeneous publication and update practices rather than systematically longer delays or reduced acquisition stability.

### 4.3. Cross-Source Harmonization and Overlap Analysis

**Figure 4:**
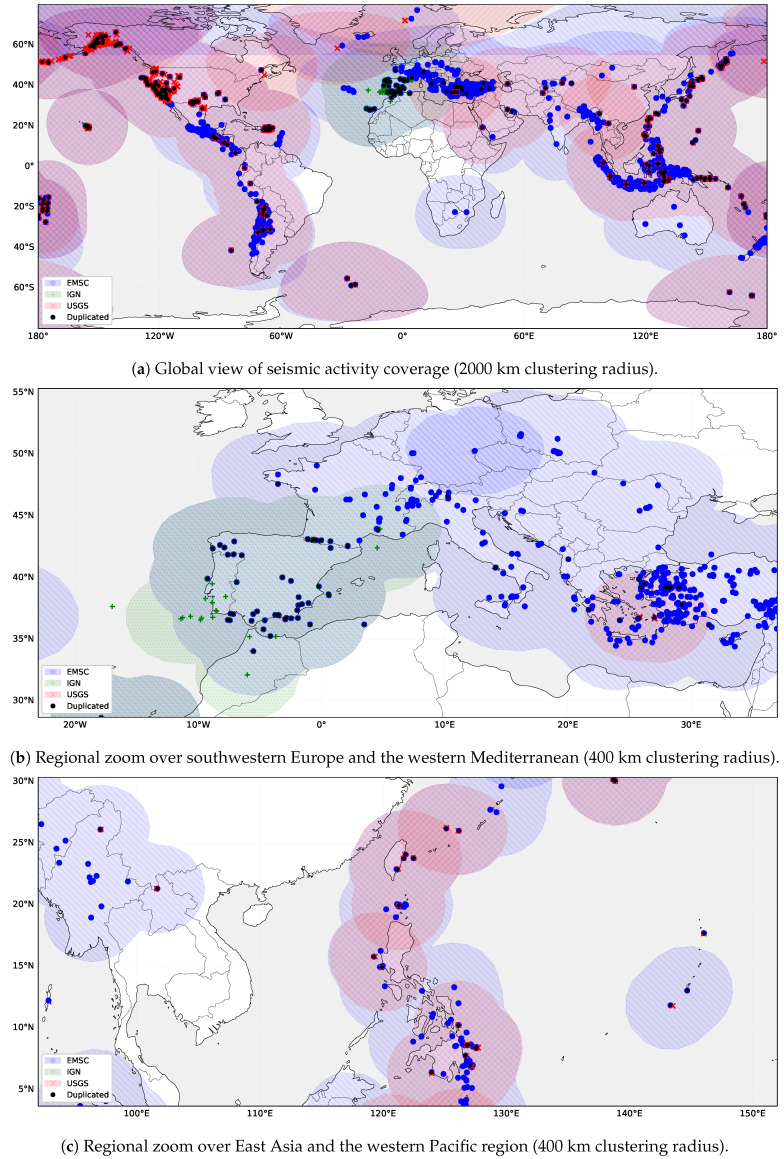
Seismic activity coverage by source catalogs during a seven-day period. Colored regions represent clustered epicentral areas derived from the USGS (red, right-striped), EMSC (blue, left-striped), and IGN (green, dotted) datasets. Overlapping regions indicate areas jointly monitored by multiple sources, while black dots denote detected duplicated earthquakes.

Following the automated harmonization and metadata enrichment, the combined event records were cross-validated across sources to evaluate spatial and temporal consistency. Figure 4a illustrates the global distribution of detections accumulated during the continuous seven-day observation period described in Section 4.2. Colored regions represent aggregated epicentral domains for each provider, obtained by clustering newly ingested events within a 2000 km radius to emphasize large-scale coverage patterns rather than individual epicenter locations.

Despite differences in declared monitoring scopes, the resulting distributions exhibit substantial geographical overlap across major tectonic zones. These overlapping regions indicate areas where the same seismic events are independently reported by multiple providers, particularly along active plate boundaries. This redundancy reflects the complementary nature of the integrated catalogs and motivates the need for systematic harmonization to ensure a coherent and non-redundant unified dataset.

Figure 4b presents a regional zoom over the western Mediterranean. The inclusion of the national IGN source increases local event density and reveals both duplicated detections and additional events not reported by larger international providers such as the EMSC. Figure 4c shows a zoom over East Asia, where a high degree of spatial coincidence is observed between events reported by the USGS and those detected by the EMSC, indicating strong overlap between the two catalogs in this region.

To complement this large-scale overlap analysis, a finer-grained comparison was conducted using data from the high-frequency 24 h validation experiment. This analysis enables a precise characterization of inter-source temporal, spatial, and magnitude discrepancies. For every possible inter-source pair, the temporal difference (Δt), epicentral distance (Δd), and magnitude difference (ΔM) were computed to identify potential duplicate reports. To reduce computational load, only event pairs within broad preliminary limits (Δt≤600 s, Δd≤50 km, ΔM≤1.0) were retained for detailed analysis, ensuring that only physically plausible coincidences were considered.

From this subset, the empirical distribution of inter-source differences was evaluated to estimate their natural variability (Table 4). Most coincident detections differed by only a few seconds and kilometers, with median offsets of 0.05 s in origin time, 0.14 km in location, and 0.02 units in magnitude. Rounded thresholds of Δt≤8 s, Δd≤8 km, and ΔM≤0.7 were adopted as representative of the 95th percentile of these distributions and used as operational limits for automatic duplicate identification. Applying these thresholds during the 24 h validation experiment yielded seven inter-source event pairs. Six of them showed close agreement within a few seconds in origin time and a few kilometers in location. One pair exhibited a moderate magnitude difference (ΔM=0.9).

A quantitative inspection of the filtered catalogs highlights notable differences in reporting conventions across providers. In the USGS catalog, only a small fraction of events exhibit negative depth values (32 events, approximately 1.8%), and a limited number report zero depth (14 events). Negative or zero magnitude values are rare, affecting fewer than 5% of reported earthquakes. In contrast, the EMSC catalog shows a markedly different pattern, with the majority of events (2554 out of 2596, approximately 98%) reporting negative depth values. A small number of events report zero depth (39 events), while no negative or zero magnitudes are observed. The IGN catalog exhibits yet another reporting pattern, with no negative depth values and a limited number of events reported at zero depth (21 events), and no anomalous magnitude values.

## 5. Discussion

The following discussion places the experimental results presented in Section 4 within the context of existing international seismic data infrastructures and large-scale earthquake catalogs. This section examines the observed operational behavior of the system in relation to existing cataloging practices. The discussion highlights consistencies with established dissemination workflows and identifies novel insights related to automation, interoperability, and continuous cross-provider integration.

### 5.1. Operational Constraints and Latency

The framework demonstrates stable near-real-time integration of seismic data from three major open sources, maintaining ingestion latencies consistently below one second and near-perfect availability during a continuous 24 h test. However, the effective timeliness of the unified catalog is constrained by the publication schedules of the source networks themselves, with typical publication delays on the order of tens of minutes, depending on provider-specific revision practices. As a result, the pipeline operates faster than the data it consumes.

In contrast to traditional global catalogs, where publication latency is attributed to post-event review workflows [35], the present results quantify how this latency manifests in modern API-driven dissemination. The median publication delay observed in this study (approximately 29 min) is consistent with workflows documented for global compilations such as ISC-GEM, where manual review, multi-agency reconciliation, and iterative revision dominate overall latency [36]. Accordingly, these findings show that end-to-end availability is structurally constrained by upstream publication and revision workflows, which are external to the proposed system. Under this operating model, further reductions in overall latency would not result from architectural optimization alone, but would require access to earlier-stage detections or alternative dissemination channels beyond formally reviewed releases.

Importantly, most of these extreme delays correspond to unique records (76.9%), indicating that they represent additional seismic information that would not be captured through other sources. Rather than reflecting deficiencies in the acquisition framework, these delayed publications extend the temporal coverage of the unified catalog by incorporating events that would otherwise remain absent. Consequently, while the IGN contributes fewer events and exhibits greater variability in publication latency, its inclusion enhances catalog completeness without compromising overall system stability.

Taken together, these results confirm the robustness and continuity of the acquisition pipeline under realistic operational conditions. They indicate that the effective publication cadence of all sources operates on time scales of several minutes. Under these conditions, a one-minute polling interval is sufficient to ensure complete event coverage without data loss, while substantially reducing network load and enabling sustainable long-term operation. This behavior is consistent with existing large-scale infrastructures such as EPOS [16] and FDSN-based services [13], which prioritize data reliability and curation over ultra-low-latency dissemination, and therefore operate on publication time scales of minutes rather than seconds.

### 5.2. Deduplication Robustness and Catalog Consistency

The observed level of cross-catalog overlap reflects the inherent redundancy of multi-agency global earthquake monitoring, where the same event is independently reported by multiple networks using distinct processing workflows. Similar reconciliation challenges are well documented in global compilations such as ISC-GEM, which rely on extensive manual effort to identify and homogenize redundant reports [36].

The adopted deduplication thresholds of 8 s, 8 km, and 0.7 magnitude units, derived from the 95th percentile of the offsets observed during the 24 h validation experiment, effectively consolidate redundant reports while preserving independent event determinations. However, this fixed-threshold approach entails inherent limitations, as inter-catalog discrepancies and scale-dependent variability are well documented in the seismological literature [37,38]. Large-magnitude earthquakes with complex rupture processes often exhibit increased variability in reported source parameters across different networks, reflecting well-known effects of rupture extent and source characterization [39]. In such cases, a static matching criterion may be overly restrictive. An adaptive deduplication strategy that incorporates event magnitude or tectonic setting could further enhance robustness, particularly in subduction zones, where location uncertainties frequently exceed ten kilometers. The observed inter-catalog discrepancies (Table 4) are consistent with typical variability reported in independent automatic catalogs [37,38]. These differences support the interpretation that the matched events represent independent detections of the same earthquakes rather than distinct events. Overall, the cross-source validation confirms the robustness of the harmonization and deduplication process, enabling near-real-time consolidation of redundant detections while preserving provenance and integrity.

Existing tools such as ObsPy require manual scripting for each data source and lack built-in deduplication. Similarly, SeisComP excels in single-network real-time acquisition but rarely supports multi-agency harmonization. In contrast, the proposed system introduces an automated, containerized pipeline that unifies USGS, EMSC, and IGN feeds while providing provenance tracking FAIR-compliant structuring. Its multi-container *Docker Compose* environment with a *PostgreSQL-PostGIS* backend supports scalable ingestion, spatial indexing, and persistent storage, while an auxiliary backup container ensures data preservation and recovery. This architecture eliminates time-consuming ad-hoc integration steps. It produces a reproducible, interoperable catalog that is immediately usable for analytical workflows and near-real-time visualization via a *GeoJSON REST API*.

### 5.3. Scalability and Data Quality

Because each data provider is handled independently within the acquisition workflow, extending the framework to additional providers would primarily increase storage requirements and network traffic in proportion to the volume of reported events. As the number of integrated providers grows, the completeness of the unified earthquake catalog would increase, but the marginal gain of newly reported unique events is expected to diminish due to increasing overlap and duplicate detections across sources, a behavior inherent to large-scale integrated seismic catalogs, where substantial effort is devoted to reconciling and harmonizing reports from multiple contributing agencies, as exemplified by the ISC-GEM compilation [36]. Consequently, while the framework can technically scale to a larger number of providers, practical deployments benefit from selecting a limited set of complementary sources rather than indiscriminately integrating a large number of redundant feeds.

From a database perspective, the use of *PostgreSQL-PostGIS* provides a mature and well-established foundation for handling concurrent spatial queries and insert operations. Observed CPU and memory usage during continuous operation remain low. This suggests that moderate increases in parallel acquisition processes, including the addition of new data providers, can be supported without architectural changes or specialized hardware (Appendix A.5).

Regarding data quality control, the proposed framework does not attempt to reinterpret or correct physical parameters reported by upstream providers, as these values originate from authoritative seismic agencies. Instead, basic filtering is applied at ingestion time to ensure semantic consistency of the integrated catalog. In particular, both the USGS and the EMSC include an explicit event-type field that distinguishes earthquakes from other seismic or non-seismic phenomena, such as explosions, quarry blasts, or avalanches. These non-earthquake events may legitimately exhibit atypical characteristics, including positive depth values or unconventional magnitude assignments. For this reason, the system restricts ingestion to confirmed earthquake-related event types only. In the case of the USGS, only events explicitly labeled as *earthquake* are retained. For the EMSC, filtering is applied to include only events classified as *ke* (known earthquake) and *fe* (felt earthquake) (*ke* and *fe* correspond to confirmed and felt earthquakes, respectively, as defined by the EMSC event classification scheme). All other event types are excluded from the unified catalog. This semantic filtering step reduces the propagation of non-comparable events across sources and improves the internal consistency of the integrated dataset.

These observations confirm that apparent numerical anomalies in depth or magnitude fields are strongly provider-dependent and often arise from differing data representations rather than data quality issues. Consequently, the proposed framework preserves all numerical values as reported by the original sources and avoids numerical thresholding or correction. Instead, consistency is ensured through semantic filtering of event types, while full provenance and reproducibility of the integrated catalog are maintained.

### 5.4. Operational Robustness

The proposed framework is designed to support continuous operation over extended periods while maintaining consistency across acquisition cycles. During the initial execution, the system performs a synchronization phase that retrieves all events available within the temporal range exposed by each provider API. Subsequent executions rely on persistent state tracking, allowing for the system to resume acquisition from the last successfully completed cycle rather than restarting from a fixed time window. As a result, if the system is stopped and resumed days or weeks later, it retrieves only the events published since the last successful acquisition. This behavior assumes that the upstream APIs provide retrospective access to previously published data. During the experimental evaluation period, all integrated provider APIs remained continuously available, resulting in 100% observed uptime.

The framework is also resilient to temporary upstream outages. If a data provider becomes unavailable for one or more acquisition cycles, the system records the failure and continues polling at the configured interval. Once the provider becomes available again, all events published during the outage window are retrieved in subsequent cycles. For example, if a provider becomes unavailable for several consecutive days and is later restored, the system automatically recovers the missing data. Recovery is performed by querying the provider from the timestamp of the last successful acquisition to the current time. This mechanism ensures that transient network failures or service interruptions do not result in permanent data loss. Temporary increases in acquisition latency may still occur, in accordance with design principles commonly adopted in resilient digital ecosystems and event-driven data infrastructures [30].

### 5.5. Limitations

These achievements are mitigated by practical constraints. The absence of an official API for the IGN forces reliance on parsing embedded JavaScript from its public portal. This approach is inherently fragile and sensitive to website redesigns or temporary service interruptions. Geographic coverage, although broad, remains incomplete at the global scale, with limited representation in parts of Africa and in some regions of the Pacific characterized by high seismic activity. Magnitude values are preserved as reported, without inter-scale homogenization. This choice may introduce systematic offsets of about 0.2 units between local and moment magnitudes.

Despite these limitations, the framework establishes an open and production-ready foundation for federated seismic data management. Its modular Docker architecture allows for seamless deployment as a microservice within emerging environmental data spaces or the EOSC, where it can act as a harmonization node for regional networks. The resulting catalog improves completeness in overlapping monitoring domains. At the same time, it preserves the traceability and interoperability required for long-term scientific reuse. This approach helps transform a historically fragmented data ecosystem into a unified, machine-actionable resource for seismological research and near-real-time applications.

## 6. Conclusions and Future Work

This work presents a modular and automated framework for the near-real-time integration of seismic data from heterogeneous open sources. The system unifies information from the USGS, EMSC, and IGN, automatically harmonizing, validating, and storing seismic events in a standardized structure. By maintaining data provenance, traceability, and interoperability, the proposed approach advances the practical implementation of the FAIR principles in seismological data management. All source code and deployment files are openly released to ensure full reproducibility and transparent reuse.

The main results of this study can be summarized as follows:

- The framework demonstrates stable and robust operation under continuous acquisition, maintaining 100% availability across all integrated providers during both short-term (24 h) and extended (7 day) deployments.
- High-frequency polling experiments (3 s interval) characterize the upper bounds of ingestion performance and confirm sub-second system latency. Long-term operation with a one-minute polling interval achieves reliable and complete ingestion without observable loss of events.
- The effective timeliness of the unified catalog is governed by provider-specific publication practices rather than by system performance, with publication delays typically on the order of a few tens of minutes, while a small number of late revisions produce a long-tailed distribution.
- Automated deduplication and harmonization successfully consolidate redundant detections across providers, reducing catalog redundancy while preserving independent event reports and metadata.
- The inclusion of sources with heterogeneous publication behaviors, such as the IGN, improves catalog completeness by incorporating late but unique events that would otherwise remain absent, without compromising system stability.

Future developments will extend the framework toward interactive and data-driven capabilities. A web-based geospatial interface will enable dynamic mapping and temporal exploration of seismic activity in real time, improving accessibility and transparency for both researchers and the general public. An alerting module will provide earthquake notifications through web and mobile applications. This component will create a lightweight early-warning channel that complements institutional systems. In parallel, *Artificial Intelligence* (AI) methods will be explored for automated tsunami risk classification and anomaly detection to enhance situational awareness and data-driven hazard assessment.

In addition to these functional extensions, further developments will focus on improving the spatial representativeness and analytical robustness of the integrated catalog. New seismic data providers will be incorporated to increase coverage in regions that are currently under-represented, particularly Africa with sources as AfricaArray [40]. Expanding the set of integrated sources is expected to improve global completeness while preserving the modular and source-agnostic design of the acquisition pipeline. As the number of integrated providers grows, further developments will also address cross-source harmonization. Beyond the fixed-threshold duplicate identification strategy evaluated in this study, adaptive and probabilistic deduplication techniques will be investigated. These methods will account for event magnitude, tectonic context, and provider-specific uncertainty characteristics, and are expected to improve robustness in complex seismic sequences and densely monitored regions.

Although the current architecture relies on centralized API integration, future work will aim to align it with emerging data-space paradigms. In these paradigms, seismic sensors, repositories, and analytical services operate as autonomous yet interoperable nodes under data sovereignty principles. This evolution would position the framework within the broader ecosystem of federated environmental and geospatial data spaces [12], promoting long-term interoperability, transparency, and reproducible open science.

## Figures and Tables

**Table 1:** Summary of access characteristics and variable nomenclature mapping across the integrated seismic data sources.

	Feature/Variable	USGS	EMSC	IGN
**General** **Characteristics**	**Geographical coverage**	Global	Europe–Mediterranean	Spain and adjacent areas
	**Access type**	Public API	Public API	Web extraction
	**Data format**	GeoJSON	JSON (GeoJSON-like)	Embedded JSON (GeoJSON-like)
**Variable Mapping** **(Unified Schema)**	**Event ID**	id	properties.unid	properties.evid
	**Origin Time**	properties.time	properties.time	properties.fecha
	**Longitude**	geometry.coordinates[0]	geometry.coordinates[0]/properties.lon	geometry.coordinates[0]
	**Latitude**	geometry.coordinates[1]	geometry.coordinates[1]/properties.lat	geometry.coordinates[1]
	**Depth**	geometry.coordinates[2]	geometry.coordinates[2]/properties.depth	properties.depth
	**Magnitude**	properties.mag	properties.mag	properties.mag
	**Magnitude Type**	properties.magType	properties.magtype	properties.magtype
	**Location/Region**	properties.place	properties.flynn_region	properties.loc
	**Source Network/Authority**	properties.net	properties.auth	—
	**Event Type**	properties.type	properties.evtype	—
	**Last Update**	properties.updated	properties.lastupdate	—
	**Source Catalog/Identifier**	properties.sources	properties.source_catalog	—
	**Reporting Network/Authority**	properties.net	properties.auth	—
	**Intensity/Felt Info**	properties.mmi	—	properties.intensidad
	**Tsunami Flag**	properties.tsunami	—	—
	**ShakeMap Flag**	properties.types (contains shakemap)	—	—

**Table 2:** Summary of 24 h acquisition performance per seismic data source. One polling cycle was executed every 3 s (28,705 iterations in ≈24 h), plus an initial synchronization cycle excluded from the statistics.

Source	New Events	Publication Delay (min)	Updated Events	Updates/Event	Availability (%)	Latency (s)	Event Rate (min^−1^)	Event Rate (cycle^−1^)
USGS	50	29.37 ± 294.18	198	1.48 ± 1.33	99.983	0.214 ± 0.441	0.035 ± 0.895	0.00174 ± 0.04492
EMSC	68	20.73 ± 156.31	128	1.63 ± 0.88	100	0.673 ± 0.109	0.047 ± 0.969	0.00237 ± 0.04861
IGN	9	37.16 ± 34.29	–	–	99.997	0.099 ± 0.085	0.006 ± 0.353	0.00031 ± 0.01770
Total	127	–	326	–	–	–	–	–
Mean	–	29.1 ± 216.12	–	1.55 ± 1.10	99.993	0.329 ± 0.304	0.088 ± 0.021	0.0044 ± 0.0011

**Table 3:** Summary of seven-day acquisition performance per seismic data source. One polling cycle was executed every 60 s (10,081 iterations over ≈7 days, ≈1440 cycles in 24 h), excluding the initial synchronization cycle.

Source	New Events	Updated Events	Duplicated Events	Publication Delay (min)	Availability (%)	Event Rate_new_ (cycle^−1^)	Event Rate_upd_ (cycle^−1^)	Event Rate_dup_ (cycle^−1^)
USGS	1816	2666	0	33.68 ± 388.58	100.0	0.180 ± 0.473	0.264 ± 0.613	0.000 ± 0.000
EMSC	2593	1067	593	24.43 ±241.31	100.0	0.257 ± 0.619	0.106 ± 0.356	0.059 ± 0.256
IGN	124	–	2	29.49 ± 631.72	100.0	0.012 ± 0.116	–	0.000 ± 0.014
**Total**	4533	3733	595	–	–	–	–	–
**Mean**	–	–	–	29.2 ± 330.87	100.0	0.450 ± 0.827	0.370 ± 0.727	0.059 ± 0.257

**Table 4:** Statistical summary of inter-catalog discrepancies in origin time (Δt), epicentral distance (Δd), and magnitude (ΔM) among coincident seismic events detected by multiple sources during the 24 h monitoring period.

Parameter	Mean	SD	Median	90%	95%	Max
Δt (s)	1.81	4.44	0.05	5.02	8.45	11.87
Δd (km)	2.58	3.56	0.14	6.89	7.93	8.97
ΔM	0.17	0.33	0.02	0.53	0.71	0.88

## Data Availability

All source code, *Docker* configurations, and deployment files are publicly available at https://github.com/Josemelgahez/global-seismic-catalog, while processed data supporting the findings of this study are available on request from the authors of this paper.

## Appendix A. Deployment and Usage Guide

This appendix describes the deployment procedure and usage workflow of the proposed seismic data integration framework. The complete implementation, including source code, configuration files, and deployment scripts, is publicly available in a dedicated GitHub repository https://github.com/Josemelgahez/global-seismic-catalog. The system is distributed as a containerized application to ensure reproducibility, portability, and ease of adoption across heterogeneous computing environments.

### Appendix A.1. Requirements

The framework requires Docker and Docker Compose to be available on the host system. The deployment procedure is platform-independent at the application level, with the following operating-system-specific considerations:

- **Windows and macOS**: Docker Desktop must be installed and running prior to executing any Docker Compose commands.
- **Linux**: Docker Engine must be installed, and the *docker* service must be running before deployment.

### Appendix A.2. Deployment

The system can be deployed locally by cloning the public repository and building the Docker containers using Docker Compose. The deployment procedure consists of the following steps:

1. **Clone the repository.**
  git clone https://github.com/Josemelgahez/global-seismic-catalog.git
  cd global-seismic-catalog
2. **Build and start the containers.**
  docker compose up --build

This command builds the required container images and launches all services defined in the *Docker Compose* configuration, including the data acquisition service, the *PostgreSQL-PostGIS* database, and auxiliary components such as the automated backup service.

### Appendix A.3. Usage

Once deployed, the system operates fully autonomously. A background acquisition scheduler continuously synchronizes with the supported seismic data providers (USGS, EMSC, and IGN), ingesting newly published or updated seismic events without requiring any user intervention.

The unified seismic catalog is accessible through the following interfaces:

- **REST API endpoint:** http://localhost:8000/api/.
- **Administrative interface:** http://localhost:8000/admin/. Default credentials: username = *admin*, password = *admin*.

The REST API provides programmatic access to the harmonized catalog, enabling integration with external analysis pipelines, visualization tools, or downstream processing workflows. The administrative interface allows for the inspection of ingested seismic events, monitoring of synchronization cycles, and verification of system status through a web-based interface.

### Appendix A.4. Data Export

The unified seismic catalog can be exported directly from the running system using standard Django management commands. This functionality supports reproducibility, offline analysis, archival, and integration with external research workflows.

The data export procedure consists of the following steps:

1. **Access the application container.**
  docker exec -it seismic-app bash
2. **Export database models to JSON format.**
  Seismic events and acquisition logs can be exported using the dumpdata command:
  python manage.py dumpdata api.Earthquake > /app/data/earthquakes.json
  python manage.py dumpdata api.CycleLog > /app/data/cycle_logs.json
  Additional database models can be exported following the same pattern:
  python manage.py dumpdata api.<ModelName> > /app/data/<filename>.json

All exported files are written to the /app/data/ directory inside the container, which is mapped by default to the ./data/ directory on the host system. This mapping enables direct access to the exported datasets from the host system, facilitating downstream analysis without additional configuration steps.

### Appendix A.5. Resource Consumption

During the seven-day continuous operation, the Docker-based deployment exhibited modest resource consumption. CPU utilization remained below 1% across all containers under normal operation, while memory usage stabilized at approximately 230 MB for the acquisition service and 150 MB for the *PostgreSQL-PostGIS* database; the backup service required less than 25 MB. During this period, the *PostgreSQL* database grew to approximately 16.8 MB, corresponding to an average growth rate of about 2.4 MB per day. This linear and modest increase, together with the low computational footprint observed, indicates that the proposed framework can be deployed on commodity hardware. Long-term operation can be sustained with minimal computational and storage requirements.

The container images used in the deployment have a fixed size of approximately 1.03 GB each, corresponding to the acquisition and backup services. This storage footprint represents a one-time deployment cost and remains constant during operation, while all dynamic data growth is confined to the database volume.
